# Preferences for Internet-Based Mental Health Interventions in an Adult Online Sample: Findings From an Online Community Survey

**DOI:** 10.2196/mental.7722

**Published:** 2017-06-30

**Authors:** Philip J Batterham, Alison L Calear

**Affiliations:** ^1^ Centre for Mental Health Research Research School of Population Health The Australian National University Acton ACT Australia

**Keywords:** Internet interventions, mental health services, preferences, anxiety, depression

## Abstract

**Background:**

Despite extensive evidence that Internet interventions are effective in treating mental health problems, uptake of Internet programs is suboptimal. It may be possible to make Internet interventions more accessible and acceptable through better understanding of community preferences for delivery of online programs.

**Objective:**

This study aimed to assess community preferences for components, duration, frequency, modality, and setting of Internet interventions for mental health problems.

**Methods:**

A community-based online sample of 438 Australian adults was recruited using social media advertising and administered an online survey on preferences for delivery of Internet interventions, along with scales assessing potential correlates of these preferences.

**Results:**

Participants reported a preference for briefer sessions, although they recognized a trade-off between duration and frequency of delivery. No clear preference for the modality of delivery emerged, although a clear majority preferred tailored programs. Participants preferred to access programs through a computer rather than a mobile device. Although most participants reported that they would seek help for a mental health problem, more participants had a preference for face-to-face sources only than online programs only. Younger, female, and more educated participants were significantly more likely to prefer Internet delivery.

**Conclusions:**

Adults in the community have a preference for Internet interventions with short modules that are tailored to individual needs. Individuals who are reluctant to seek face-to-face help may also avoid Internet interventions, suggesting that better implementation of existing Internet programs requires increasing acceptance of Internet interventions and identifying specific subgroups who may be resistant to seeking help.

## Introduction

There has been rapid growth in the number and variety of Internet-based interventions targeting mental health problems in the community since the emergence of pioneering programs such as MoodGYM approximately 15 years ago. Evidence for Internet interventions (also referred to as “online programs”) has also grown rapidly. Several meta-analyses have been conducted and report considerable evidence of effectiveness for programs to treat depression [1], anxiety [2], and substance use disorders [3,4], with emerging evidence of effectiveness for reducing suicidal ideation [5-7]. There is also evidence that such programs can be beneficial in a prevention setting [8,9]. However, there has been limited effort to disentangle the attributes of programs that are associated with better outcomes beyond broad categories such as clinician guidance and length of program. Moreover, the development and implementation of such programs is rarely guided by the preferences of those in the community who may use them. By taking into account preferences in the community for the use of online programs, it may be possible to increase uptake and increase adherence, leading to better outcomes.

Internet-based mental health interventions have typically been modeled on a psychological therapy format. Most are based on some form of cognitive and/or behavioral therapy, in which 50-minute weekly consultations over 6 to 10 weeks are the norm. Consequently, lengthy weekly modules tend to have been adopted for Internet interventions. This structure is designed to allow users to learn and practice new skills over the week. However, much content on the Internet is not designed in this way, with users spending no more than 70 seconds on 80% of Web pages [10] and average YouTube videos lasting less than 5 minutes [11]. Therefore, it might be reasonable to infer that users of Internet interventions may have a preference for a different model of engagement with online programs than for face-to-face therapy.

User preferences may also influence other aspects of the design of Internet interventions. Preferences for modality of content, with choices including video (live action or animated), text, images, or a combination, might be associated with learning styles and impact on engagement with online content [12,13]. Characterizing these preferences in a community-based setting can consequently inform design choices for the development of new interventions. There has been limited research exploring user preferences for Internet interventions. A recent study by McClay and colleagues [14] in the area of online interventions for eating disorders has indicated that users had a preference for weekly engagement and sessions of 20 minutes or less. In a study of women with postpartum depression, 87.5% indicated a preference for intervention sessions of 15 to 30 minutes, whereas a third of women wanted videos to illustrate ways to cope and 65% wanted a chat room that was moderated by an expert in postpartum depression [15]. In examining preferences of young adults with first-episode psychosis, Lal et al [16] reported that a mixture of modalities (video, text, images) was preferred, whereas in a study of preferences for alcohol and drug websites, high-cost features (eg, videos, animations, and games) were less highly valued than website design/navigation, being open access, having validated content, and the option for email therapist support [17]. The relatability of content, a preference for action-based rather than talk-based therapies, and opportunities to build skills were highlighted as preferences for online mental health services by young men [18]. Few other studies have identified optimal frequency, duration, or modality of support.

Underlying these preferences is the assumption that individuals are willing to engage in online therapy. A number of studies have reported a greater preference for face-to-face therapy [17,19-21]. For instance, a study by Casey and colleagues [19] reported that even though participants perceived fewer barriers to online therapy, they still reported a greater intention to access face-to-face therapy. Identifying preferences for engagement with online versus face-to-face therapy is a key question in determining the scope for broader uptake of Internet interventions in the community. Disadvantages of online therapy identified in previous research have included concerns about their helpfulness and credibility, suitability (low computer literacy), personability, and confidentiality, whereas advantages have included flexibility (time and location), accessibility, anonymity, user empowerment, and low effort [22-24]. Furthermore, testing predictors of preferences for online programs can assist in identifying subpopulations where engagement may be challenging. For instance, in a study of adolescent preferences for mental health services, male participants were 1.7 times more likely to prefer online services than female participants [24]. In another study of preferences, participants who preferred eHealth services were found to have higher stigmatized beliefs and lower scores on extraversion, agreeableness, emotional stability, and openness to experience than those who preferred traditional services [17]. Other factors reported to be associated with willingness to participate in an online intervention may include older age, female gender, being separated or divorced, being highly educated, history of depression, and lower levels of personal stigma and higher levels of depressive symptoms [25].

This study aimed to survey preferences for Internet-based mental health interventions in an online Australian sample. Specifically, the study aimed to determine optimal duration and frequency of modules or sessions, and whether there was a trade-off between duration and frequency or content. Preferences for modality of content were also examined to determine whether there was clear support for a particular format or platform for presenting therapeutic content and psychoeducation. Preferences for face-to-face versus Internet treatment were independently assessed, and predictors of these preferences were tested to identify subgroups where engagement in online programs may be particularly challenging. The questions used in the study were informed by the Discrete Choice Experiment approach [26,27]; however, the large number of attributes and potential attribute levels restricted the focus to only the main effects of each attribute of interest, ensuring response burden was not excessively onerous. The findings from this study may inform the development of new online mental health programs and better dissemination of existing programs.

## Methods

### Participants and Procedure

Australian adults were recruited on the social media platform Facebook between August 2014 and January 2015. Advertisements included the text “Complete a 20-30 min survey to help us develop online services for mental health” and the logo of the Australian National University (ANU). To enable comparison between young adults and older adults, purposive oversampling of those aged between 18 and 25 years was conducted, using targeted advertising to this age group. Advertisements linked directly to an online survey that included questions on preferences for Internet-based programs, Internet usage, and mental health status, with a separate set of questions on help seeking for suicidal thoughts that were not included in the current analyses. Individuals could also engage with the study through a Facebook page, enabling sharing of the study through social networks. The survey was implemented online using LimeSurvey, with data stored on a secure server at ANU. From 859 individuals who engaged with the survey link, 673 (78.3%) respondents consented to participate in the study and 438 (50.9%) adults fully completed the survey and were included in these analyses. The study received ethical approval from the ANU Science and Medical Delegated Ethical Review Committee (protocol #2014/380, approved July 23, 2014).

### Measures

Internet-based programs were defined for participants as websites that provided effective strategies to change unhelpful thinking patterns, effective strategies to change unhealthy lifestyle patterns, or effective strategies for relaxation. Interest in potential components of an Internet-based program was assessed based on perceived helpfulness (rated on a 5-point scale from “very unhelpful” to “very helpful”) and appeal (rated on a 5-point scale from “very unappealing” to “very appealing”). Components queried included screening scales to assess mental health, feedback about mental health symptoms, information about mental health problems (eg, signs, symptoms, risk factors, treatments), strategies to change unhealthy lifestyles, strategies to change unhelpful thoughts, and negative feelings and relaxation strategies.

Preferences for duration and frequency of delivery of online mental health programs were assessed using four questions comparing different delivery scenarios based on low- or high-intensity programs and testing preferences for frequency or duration. The scenarios were chosen to probe preferences for brief versus long sessions (duration) and frequent versus extended sessions (frequency), reflecting the typical demands of existing Internet-based programs:

1. One 2-hour session on 1 day (ie, once-off long session) versus ten 12-minute sessions across 10 days (ie, multiple brief sessions)
2. Five 60-minute sessions over 5 weeks (ie, high-intensity weekly sessions) versus fifteen 20-minute sessions over 3 weeks (ie, moderate-intensity high-frequency sessions)
3. Fifteen 20-minute sessions over 3 weeks (ie, moderate-intensity high-frequency sessions) versus ten 10-minute sessions over 10 weeks (ie, extended period of brief weekly sessions)
4. Five 60-minute sessions over 5 days (ie, intensive sessions over a short period) versus five 60-minute sessions over 5 weeks (ie, intensive sessions over a long period)

In addition, willingness to participate in a 10-minute daily session or 50-minute weekly session was assessed by asking how many days/weeks participants would continue within such a program.

Preference for tailored versus generic programs was also assessed using a single item, comparing “a program that is tailored to your needs and preferences, that would take a little longer to assess your needs and preferences” to “a general program that is the same for everyone, without the need to assess needs and preferences.” All preference items had a “no preference” response option.

Preferences for modality of online content delivery were based on items assessing preference for reading text (information) online, reading text (story) online, watching an animated video, watching a live action video, and looking at images/graphics. Each modality was rated on 5-point scale from “strongly avoid” to “strongly prefer.” Similarly, preferences for setting of online content delivery were assessed using six items referring to hardware and location, each rated on a 6-point scale from “don’t use” to “strongly prefer”: smartphone at home, smart phone at work/school, laptop/desktop at home, laptop/desktop at work/school, tablet at home, and tablet at work/school.

Finally, preference for online programs compared to face-to-face programs was assessed by identifying whether individuals reported being “highly likely” or “likely” to use such a program if they were having a personal or emotional problem (face-to-face programs were specified as including counseling and group programs). Participants were categorized as likely to use (1) either face-to-face or online programs, (2) only face-to-face, (3) only online, or (4) neither face-to-face nor online. Preferences for online program use were further explored by asking about four situations in which the respondent might use such programs, with usage in each scenario rated on a 5-point scale from “highly unlikely” to “highly likely”: (1) “if I wanted to increase my happiness and general well-being,” (2) “if I was at risk of developing a mental health problem,” (3) “if I was experiencing symptoms of a mental health problem,” and (4) “if I had been diagnosed with a mental health problem.”

Potential predictors of preferences included demographic characteristics (age, gender, level of education, linguistic diversity—English vs non-English—marital status, employment status), depression symptoms (Patient Health Questionnaire-9; PHQ-9 [28]), anxiety symptoms (Generalized Anxiety Disorder-7; GAD-7 [29]), suicidal ideation (Suicidal Ideation Attributes Scale; SIDAS [30]), Attitudes Toward Seeking Professional Help (ATSSPH [31]), access to broadband Internet and a device at home, and typical time spent on the Internet at school or work and home.

### Analysis

Analyses were largely descriptive, reporting interest in components of online programs, preferences for duration/frequency, modality, and setting, and comparison between online and face-to-face. Bonferroni-adjusted post hoc paired *t* tests were used to compare item responses, with corrected alphas reported in the Results section. Predictors of preferences were examined using logistic regression models and a linear regression model on number of sessions that individuals reported they would complete. All analyses were conducted in SPSS version 23 (IBM Corp, Chicago, IL, USA).

## Results

Of the 438 participants who completed the survey, the majority were female (78.5%, n=344) and the mean age was 34.9 (SD 15.5) years. By design, the group between 18 and 25 years was overrepresented (44.5%, n=195); the group between 26 and 45 years comprised 26.9% (n=118) of the sample and 28.5% (n=125) were aged 46 years or older. The sample was fairly well educated; 37.4% (164/438) did not have postsecondary education; 14.4% (63/438) completed a certificate, diploma, or associate degree; 24.4% (107/438) completed a bachelor degree; and 23.1% (101/438) completed a higher degree. In addition, 45.4% (199/438) were current tertiary (university) students. Although most respondents spoke only English at home, 11.2% (49/438) reported speaking another language. Respondents were married (37.9%, 166/438), unmarried and not in a relationship (30.8%, 135/438), unmarried and in a relationship (18.9%, 83/438) or divorced/separated (11.0%, 48/438). Most respondents were employed, either in full-time (33.1%, 145/438) or part-time (32.8%, 144/438) work, although 9.4% (41/438) were unemployed and 23.9% (105/438) were not currently in the labor force (eg, maternity leave, retired, full-time students). Mental health symptoms were elevated in the sample, with mean PHQ-9 depression score of 10.2 (SD 7.4) and GAD-7 score of 7.7 (SD 6.3), indicating moderate levels of depression and anxiety symptoms. Some level of suicidal ideation in the past month was reported by 45.8% (201/438) of participants.

### Interest in Potential Components of an Internet-Based Program

Table 1 reports the perceived helpfulness and appeal of various components or attributes of online mental health programs. Mean scores correspond to scale responses ranging from 1=“very unhelpful/unappealing” to 5=“very helpful/appealing.” Bonferroni-adjusted post hoc paired *t* tests indicated that the first two components (screening and feedback) were rated significantly less helpful than other components (*P*<.001 for all comparisons except feedback vs relaxation strategies, *P*=.19), feedback was rated as significantly more helpful than screening alone (*P*<.001), and “strategies to change unhelpful thoughts and negative feelings” was perceived as significantly more helpful than “relaxation strategies” (*P*=.002). Similarly, the first two components (screening and feedback) were rated significantly less appealing than other components (*P*<.001 for all comparisons except feedback vs changing unhealthy lifestyles, *P*=.02, and feedback vs relaxation strategies, *P*=.009), feedback was rated as significantly more helpful than screening alone (*P*<.001), and the “thoughts and feelings” item was perceived as significantly more appealing than both “lifestyle” and “relaxation” strategies (*P*<.001 for both).

### Preferences for Duration and Frequency

Participants had a preference for breaking up long sessions, with most (58.0%, 254/438) preferring ten 12-minute sessions across 10 days rather than a single 2-hour session (21.0%, 92/438; the remaining 21.0%, 92/438 had no preference). However, for programs with higher intensity, this preference faded, with 42.5% (186/438) preferring fifteen 20-minute sessions over 3 weeks and 40.0% (175/438) preferring five 60-minute sessions over 5 weeks.

There were no strong preferences for frequency of program delivery when sessions were brief: 43.2% (189/438) preferred ten 10-minute sessions over 2 weeks and 35.8% (157/438) preferred ten 10-minute sessions over 10 weeks. However, frequency preferences became stronger with longer session duration, with 65.8% (288/438) preferring five 60-minute sessions over 5 weeks compared to 13.9% (61/438) preferring five 60-minute sessions over 5 days. In summary, participants generally had a preference for sessions of shorter duration, but if longer sessions were required, they preferred that sessions be delivered less frequently.

When asked how many sessions they would be willing to complete of a 10-minute daily session, the mean response was 13.8 (SD 10.6, range 0-30) sessions. Three-quarters of participants reported willingness to complete six or more sessions. Similarly, when asked about completion of 50-minute weekly sessions, participants reported a mean of 8.1 (SD 8.7, range 0-30) sessions, with three-quarters willing to complete three or more sessions.

### Preferences for Tailoring

Participants reported a strong preference for tailored programs (tailored: 81.1%, 355/438; generic programs: 13.9%, 61/438; no preference: 5.0%, 22/438), even if that resulted in extra time required to assess needs and preferences.

### Preferences for Modality and Setting

Table 2 shows preferences for modality, rated on a 5-point scale (1=“strongly avoid” to 5=“strongly prefer”) and setting of use, rated on a 6-point scale (1=“don’t use,” 2=“strongly avoid” to 6=“strongly prefer”). Overall, none of the modalities stood out as being strongly preferred. Bonferroni-adjusted paired *t* tests indicated that informational text and images were significantly preferred over any type of video (*P*<.001 for all comparisons), and narrative text was preferred over animated videos (*P*<.001). There were stronger preferences for specific settings and hardware for use, with greater preference for completion of online mental health programs using laptop/desktop computers over tablets and mobile phones (*P*<.001 for all comparisons), greater preference for mobile phones over tablets (*P*<.001 for all comparisons), and greater preference for completing programs at home rather than at work or school for each device (*P*<.001 for all comparisons).

**Table 1:** Helpfulness and appeal of components of online mental health programs (N=438).

Component^a^	Helpfulness		Appeal	
	Mean (SD)	Helpful, n (%)	Mean (SD)	Appealing, n (%)
Screening scales to assess mental health	3.75 (0.96)	305 (69.6)	3.40 (1.02)	216 (49.3)
Feedback about mental health symptoms	3.97 (0.85)	357 (81.5)	3.66 (0.95)	295 (67.4)
Information about mental health problems	4.12 (0.82)	382 (87.2)	3.91 (0.87)	344 (78.5)
Strategies to change unhealthy lifestyles	4.11 (0.83)	376 (85.8)	3.78 (0.92)	308 (70.3)
Strategies to change unhelpful thoughts and negative feelings	4.14 (0.88)	373 (85.2)	3.94 (0.91)	342 (78.1)
Relaxation strategies	4.04 (0.90)	346 (79.0)	3.81 (0.93)	298 (68.0)

^a^ Rated on a 5-point scale (1=“very unhelpful/unappealing” to 5=“very helpful/appealing”).

**Table 2:** Preferences for modality and setting of use (N=438).

Attribute		Mean (SD)	Prefer, n (%)	First preference, n (%)
**Modality^a^**				
	Reading text (information)	3.63 (0.89)	267 (61.0)	158 (36.1)
	Reading text (story)	3.49 (0.92)	245 (55.9)	76 (17.4)
	Watching an animated video	3.19 (1.09)	194 (44.3)	65 (14.8)
	Watching a live action video	3.31 (1.09)	218 (49.8)	84 (19.2)
	Looking at images/graphics	3.61 (0.89)	281 (64.2)	55 (12.6)
**Setting^b^**				
	Mobile phone at home	3.90 (1.59)	209 (47.7)	—
	Mobile phone at work/school	3.36 (1.62)	133 (30.4)	—
	Laptop/desktop at home	5.29 (0.97)	391 (89.3)	—
	Laptop/desktop at work/school	3.81 (1.70)	199 (45.4)	—
	Tablet at home	3.29 (1.98)	168 (38.4)	—
	Tablet at work/school	2.38 (1.56)	50 (11.4)	—

^a^ Rated on 5-point scale (1=“strongly avoid” to 5=“strongly prefer”).

^b^ Rated on a 6-point scale (1=“don’t use,” 2=“strongly avoid” to 6=“strongly prefer”).

**Table 3:** Reasons for using online mental health programs (N=438).

Reason for use^a^	Mean (SD)	Likely, n (%)
If I wanted to increase my happiness and general well-being	3.04 (1.29)	187 (42.7)
If I was at risk of developing a mental health problem	3.03 (1.24)	184 (42.0)
If I was experiencing symptoms of a mental health problem	3.47 (1.24)	265 (60.5)
If I had been diagnosed with a mental health problem	3.68 (1.26)	310 (70.8)

^a^ Rated on a 5-point scale (1=“highly unlikely” to 5=“highly likely”).

### Preference for Online Programs Compared to Face-to-Face Programs

The majority (86.1%, 377/438) of participants reported that they would likely seek help from either an online or face-to-face source if they were experiencing a personal or emotional problem. More participants endorsed likelihood of using face-to-face sources only (25.3%, 111/438) than online programs only (14.4%, 63/438), although nearly half (46.3%, 203/438) reported that they would be likely to use both sources.

Four specific reasons for using online mental health programs were examined (Table 3). Participants reported that they would be most likely to use an online mental health program if they were diagnosed with a mental health problem, followed by experiencing symptoms of a mental health problem, followed by being at risk for a mental health problem and for increasing well-being, with no difference between these latter categories (*P*=.76), but significant pairwise differences otherwise (*P*<.001 for all comparisons).

### Predictors of Preferences

Tables 4 and 5 show the outcome of a series of logistic regression models examining predictors of various preferences: likely to use online programs, likely to use face-to-face programs, preference for video content, and preference for tailored content. A linear regression was also conducted to examine predictors of number of daily 10-minute sessions the individual reported they would complete. Younger age was associated with higher preference for online and lower preference for face-to-face programs. Females were more likely to report a preference for online programs. More education was associated with greater preference for online programs and greater preference for video content. Participants who were unmarried were less likely to have a preference for online programs, although they reported that they would complete more sessions of an online program than those who were married. Participants with more favorable attitudes toward seeking professional help had greater preference for both online and face-to-face programs, greater preference for tailored programs, and reported that they would complete more sessions of an online program. Internet availability and usage were not associated with preferences, although those with a broadband connection at home were less likely to have a sole preference for face-to-face programs. Finally, mental health symptoms had no relationship with preferences for online programs.

**Table 4:** Logistic and linear regression models of predictors of preferences for online mental health programs: likely to use online and likely to use face-to-face.

Independent variable		Likely to use online		Likely to use face-to-face	
		OR (95% CI)	*P*	OR (95% CI)	*P*
Age		0.98 (0.95, 1.00)	.04	1.04 (1.01, 1.07)	.01
Gender=female vs male		2.02 (1.21, 3.36)	.007	0.91 (0.48, 1.73)	.78
**Education**			.02		.20
	Certificate/Diploma/Associates	2.83 (1.37, 5.85)	.005	0.89 (0.35, 2.24)	.80
	Bachelor	1.71 (0.98, 2.99)	.06	0.88 (0.45, 1.72)	.71
	Higher degree	2.02 (1.06, 3.85)	.03	0.44 (0.19, 0.98)	.046
	High school or less (reference category)	1.00		1.00	
Speak English only at home		0.78 (0.39, 1.55)	.47	0.55 (0.24, 1.26)	.16
**Marital status**			.002		.50
	Unmarried, no partner	0.34 (0.16, 0.71)	.004	0.70 (0.30, 1.63)	.41
	Unmarried, partnered	0.32 (0.17, 0.60)	<.001	0.79 (0.38, 1.64)	.53
	Divorced, separated, widowed	0.54 (0.27, 1.07)	.08	1.79 (0.65, 4.91)	.26
	Married (reference category)	1.00		1.00	
**Employment status**			.10		.68
	Unemployed	1.66 (0.76, 3.60)	.20	0.98 (0.40, 2.40)	.97
	Not in labor force	1.71 (1.01, 2.89)	.04	1.32 (0.70, 2.49)	.39
	Employed full/part time (reference category)	1.00		1.00	
Attitudes toward professional help seeking (ATSPPH-SF)		1.05 (1.01, 1.09)	.008	1.27 (1.20, 1.34)	<.001
Home Internet/hardware		0.43 (0.16, 1.14)	.09	0.17 (0.04, 0.76)	.02
Work/school Internet/hardware		1.35 (0.79, 2.30)	.27	0.89 (0.47, 1.70)	.73
Frequently use home Internet		0.74 (0.45, 1.21)	.23	0.58 (0.32, 1.03)	.06
Frequently use work/school Internet		0.70 (0.36, 1.36)	.30	1.08 (0.46, 2.50)	.87
Depression symptoms (PHQ-9)		0.99 (0.94, 1.05)	.76	0.98 (0.91, 1.04)	.48
Anxiety symptoms (GAD-7)		0.99 (0.93, 1.05)	.70	1.05 (0.98, 1.13)	.18
Suicidal ideation		0.99 (0.96, 1.01)	.31	1.00 (0.97, 1.03)	.91
Constant/intercept		2.14 (0.28, 16.31)	.46	0.01 (0.00, 0.21)	.002

**Table 5:** Logistic and linear regression models of predictors of preferences for online mental health programs: preference for video content, preference for tailored content, and number of sessions.

Independent variable		Preference for video content		Preference for tailored content		Number of sessions	
		OR (95% CI)	*P*	OR (95% CI)	*P*	Estimate	*P*
Age		1.00 (0.98, 1.02)	.97	0.98 (0.95, 1.00)	.09	9.66 (0.16, 19.16)	.55
Gender=female vs male		0.92 (0.56, 1.52)	.74	1.53 (0.86, 2.72)	.15	-0.24 (-2.69, 2.21)	.85
**Education**			.03		.45		
	Certificate/Diploma/Associates	2.61 (1.33, 5.13)	.005	0.86 (0.38, 1.91)	.71	-1.60 (-4.65, 1.45)	.30
	Bachelor	1.07 (0.60, 1.89)	.83	1.61 (0.77, 3.36)	.21	-1.19 (-4.56, 2.18)	.49
	Higher degree	1.40 (0.74, 2.64)	.30	1.24 (0.58, 2.63)	.58	1.39 (-1.62, 4.41)	.37
	High school or less (reference category)	1.00		1.00			
Speak English only at home		0.73 (0.38, 1.42)	.36	1.12 (0.50, 2.51)	.79	-2.08 (-5.30, 1.15)	.21
**Marital status**			.19		.78		
	Unmarried, no partner	0.53 (0.25, 1.12)	.10	0.67 (0.27, 1.65)	.39	-1.23 (-4.55, 2.09)	.47
	Unmarried, partnered	1.05 (0.58, 1.90)	.87	0.92 (0.43, 1.98)	.83	-5.17 (-9.58, -0.76)	.02
	Divorced, separated, widowed	1.20 (0.62, 2.35)	.59	0.80 (0.37, 1.71)	.56	-4.48 (-8.46, -0.50)	.027
	Married (reference category)	1.00		1.00			
**Employment status**			.65		.34		
	Unemployed	1.33 (0.64, 2.79)	.45	1.17 (0.43, 3.19)	.76	-0.55 (-3.02, 1.92)	.66
	Not in labor force	0.92 (0.55, 1.55)	.75	0.67 (0.36, 1.22)	.20	1.28 (-2.58, 5.14)	.52
	Employed full/part time (reference category)	1.00		1.00			
Attitudes toward professional help seeking (ATSPPH-SF)		1.02 (0.99, 1.06)	.23	1.05 (1.00, 1.10)	.05	-2.42 (-6.76, 1.93)	.006
Home Internet/hardware		0.79 (0.33, 1.92)	.61	1.58 (0.57, 4.34)	.38	-0.63 (-3.14, 1.88)	.28
Work/school Internet/hardware		0.75 (0.45, 1.26)	.28	1.21 (0.64, 2.26)	.56	-0.10 (-2.41, 2.21)	.62
Frequently use home Internet		1.17 (0.72, 1.89)	.53	1.26 (0.68, 2.31)	.46	-1.37 (-4.55, 1.80)	.93
Frequently use work/school Internet		0.52 (0.26, 1.06)	.07	1.12 (0.53, 2.37)	.77	-0.03 (-0.14, 0.07)	.40
Depression symptoms (PHQ-9)		1.02 (0.96, 1.07)	.54	1.04 (0.97, 1.11)	.30	0.26 (0.07, 0.44)	.10
Anxiety symptoms (GAD-7)		1.00 (0.95, 1.06)	.95	0.98 (0.91, 1.05)	.59	0.22 (-0.04, 0.49)	.27
Suicidal ideation		0.99 (0.96, 1.02)	.48	1.02 (0.99, 1.06)	.23	-0.15 (-0.43, 0.12)	.53
Constant/intercept		0.36 (0.05, 2.70)	.32	0.60 (0.06, 6.36)	.67	0.04 (-0.09, 0.17)	.046

## Discussion

There were a number of key findings from this study regarding the preferences of adults for delivery and usage of online mental health programs. The component of online programs that received the highest preference was “strategies to change unhelpful thoughts and negative feelings.” This finding is consistent with the cognitive and/or behavioral strategies employed by most online mental health programs and is similar to previous research that identified opportunities to build skills as a preference for online programs [18]. Although screening and feedback about symptoms were less desirable components, there was a strong preference that material be tailored to the individual, even if that requires a screening process. Participants reported a preference for briefer sessions of online programs, consistent with previous findings [14]. However, there was a trade-off between duration and frequency, with a preference for delivery of brief sessions over shorter periods, but a preference for greater time between sessions when programs included longer sessions. Participants with more positive attitudes toward seeking professional help were likely to endorse longer programs, suggesting that a more positive overall perspective on psychological treatments facilitates greater engagement with treatment programs. The desire for briefer programs may be met by the tailoring of content to individual needs [32], a greater focus on the core components of cognitive and behavioral therapies with an emphasis on overlearning [33], and delivery of activities to practice therapeutic strategies delivered offline through stand-alone worksheets or through mobile and ecological interventions [34].

There were mixed preferences for the modality of presentation, with some preferring text and others preferring images or videos. This finding suggests a mixture of modalities may be acceptable, consistent with Lal et al [16]. Alternatively, providing users with the option to choose a presentation format that suits their preferences may also increase engagement. Few predictors of modality preferences were identified, although there was some indication that increased education was associated with greater preference for video-based programs. Despite the proliferation of mobile technology in recent years, most users reported a preference for accessing online programs through a laptop or desktop computer. It may be that the description of online programs did not specifically incorporate elements of mobile health programs, which may have raised concerns about the accessibility of online programs on mobile devices. Respondents also reported a preference for accessing programs in the home rather than at work or school, which may be related to privacy concerns.

There was a fairly high acceptance of online mental health programs, with 71% reporting that they would be likely to use an online program if they were diagnosed with a mental illness and 60% if they were experiencing “emotional or personal problems” more broadly. Use of online programs for subclinical states and to enhance well-being was somewhat lower but still remained above 40%. Face-to-face treatment was slightly preferred, in line with previous research [17,19-21], with 71% reporting they would use face-to-face treatment if experiencing emotional or personal problems. There may be a number of reasons for this preference, including a lack of familiarity with the evidence supporting e-mental health programs, conflation of evidence-based e-mental health programs with non-evidence-based websites, suspicion of therapy without direct human contact, or concerns about privacy of personal information in an online setting [17].

Factors associated with greater likelihood of using online programs included younger age, female gender, increased education, being married, and positive attitudes toward professional help seeking. A number of the factors identified are consistent with those reported by Crisp and Griffiths [25] as being associated with an interest in online programs, such as being older, female, separated or divorced, highly educated, and having lower levels of personal stigma. More positive attitudes toward professional help seeking were also associated with a greater likelihood of using face-to-face programs, suggesting these attitudes reflect a general tendency for engagement with psychological treatment. Older age and poorer access to Internet in the home were also associated with greater likelihood of using face-to-face treatment, indicating a divergence between younger and older participants and that access to technology remains a potential barrier to uptake of online services. The gender differences in likely use of online interventions is interesting because many assume that males engage in face-to-face services less than females because they do not like to talk about their problems; barriers men face are likely to be more diverse [35]. These findings suggest that males may similarly require additional persuasion to engage in online programs, suggesting that more work is needed to encourage uptake among males who less typically receive help for mental health problems than females [36]. Addressing the differences in preferences based on age, gender, and education requires further research, identifying technological and structural processes required to make older and less educated individuals more comfortable with online programs. Further investigation of acceptance facilitating interventions [37] as an approach for increasing uptake of online interventions appears to be a promising avenue for bridging these divides.

Although this was one of the first studies to assess preferences for online mental health programs in the community, there were some limitations. The study assumed that participants had a shared understanding of what online programs can deliver to users. It was intended that this shared understanding be imparted by providing a definition of online programs to participants. However, preferences for use of online programs may be shaped by knowledge and attitudes toward such programs and past experience with online and face-to-face therapy. There may have been a diversity of experience, with some participants in the current study having extensive knowledge of online programs and some having no awareness before the survey. Therefore, interpretation of questions regarding preferences for use of online programs may have been entirely hypothetical for a section of the participants. Although analyses examined the role of attitudes toward professional help seeking in preferences for online programs, further examination of the roles of attitudes and knowledge regarding online programs in shaping preferences for care is warranted. Examination of additional factors associated with preferences is also encouraged because few strong predictors emerged from the regression analyses.

The sample was recruited online, which may be most appropriate for the study of preferences for online programs, although such samples may have limited representativeness of the broader community. In particular, participants had elevated mental health symptoms, probably reflecting self-selection into the study, and males were underrepresented. Although the study aimed to recruit a diverse sample, future research may benefit from targeting specific subgroups to ensure that the development of online programs takes into account diverse needs and preferences. Research on the preferences of males in particular is needed to ensure that their low usage of online (and in-person) services is not perpetuated. The study adopted elements of Discrete Choice Experiments, but the number of attributes of interest precluded a more thorough evaluation of interactions between preferences for different attributes of delivery. Further in-depth research of user priorities for different delivery attributes may provide additional insight into the optimal design of online programs. Finally, user-reported preferences may be quite different to preferences in practice (eg, it would be uncommon for users to complete eight weekly 50-minute sessions of an online intervention). In addition, the implementation of programs based on user preferences may not always have positive effects on outcome. Creating programs with the flexibility to accommodate diverse needs and preferences may be helpful for optimizing uptake and adherence.

In conclusion, this study identified preferences for components, duration, frequency, modality, and setting of online mental health programs. Developers of new programs may benefit from taking into account the preferences of potential users in the community because meeting these preferences may result in greater uptake and adherence. Furthermore, better implementation of existing programs requires identifying subgroups of the population who may be resistant to addressing mental health symptoms using the Internet. This study identified that older people, males, less educated, and unmarried people may be less likely to engage in online mental health programs, along with people who have negative attitudes toward professional psychological treatments. The assumption that individuals who do not typically engage in face-to-face treatment will necessarily prefer online treatment may be inaccurate, suggesting that engaging these groups in appropriate treatment will require innovation and better matching of treatments with individual preferences.

## Abbreviations

ANU: Australian National University
ATSSPH: Attitudes Toward Seeking Professional Help
GAD-7: Generalized Anxiety Disorder-7
PHQ-9: Patient Health Questionnaire-9
SIDAS: Suicidal Ideation Attributes Scale
